# New insights into chasmosaurine (Dinosauria: Ceratopsidae) skulls from the Upper Cretaceous (Campanian) of Alberta, and an update on the distribution of accessory frill fenestrae in Chasmosaurinae

**DOI:** 10.7717/peerj.5194

**Published:** 2018-07-03

**Authors:** James A. Campbell, Michael J. Ryan, Claudia J. Schröder-Adams, David C. Evans, Robert B. Holmes

**Affiliations:** 1 Department of Earth Sciences, Carleton University, Ottawa, ON, Canada; 2 Department of Biological Sciences, University of Calgary, Calgary, AB, Canada; 3 Department of Vertebrate Paleontology, Cleveland Museum of Natural History, Cleveland, OH, USA; 4 Royal Ontario Museum, Toronto, ON, Canada; 5 Department of Biological Sciences, University of Alberta, Edmonton, AB, Canada

**Keywords:** Campanian, *Chasmosaurus*, Dinosaur Park Formation, Laramidia, Oldman Formation, *Vagaceratops*, Squamosal, Parietal, Accessory fenestra

## Abstract

Chasmosaurine ceratopsids are well documented from the Upper Cretaceous (Campanian) Dinosaur Park Formation (DPF) of southern Alberta and Saskatchewan, and include *Chasmosaurus belli*, *Chasmosaurus russelli*, *Mercuriceratops gemini*, *Vagaceratops irvinensis*, and material possibly referable to *Spiclypeus shipporum.* In this study, we describe three recently prepared chasmosaurine skulls (CMN 8802, CMN 34829, and TMP 2011.053.0046) from the DPF, and age-equivalent sediments, of Alberta. CMN 8802 and CMN 34829 are both referred to *Chasmosaurus* sp. based on the size and shape of the preserved parietal fenestrae. TMP 2011.053.0046 is referred to *Vagaceratops* sp. based on the position and orientation of its preserved epiparietals. Each skull is characterized by the presence of an accessory fenestra in either the squamosal (CMN 8802 and TMP 2011.053.0046) or parietal (CMN 34829). Such fenestrae are common occurrences in chasmosaurine squamosals, but are rare in the parietal portion of the frill. The origin of the fenestrae in these three specimens is unknown, but they do not appear to exhibit evidence of pathology, as has been previously interpreted for the accessory fenestrae in most other chasmosaurine frills. These three skulls contribute to a better understanding of the morphological variation, and geographic and stratigraphic distribution, of chasmosaurines within the DPF and age-equivalent sediments in Western Canada.

## Introduction

Ceratopsidae represents a diverse and successful radiation of megaherbivorous, ornithischian dinosaurs. This family is known from Late Cretaceous (Campanian–Maastrichtian) sediments deposited primarily in what is now western North America, and, to a lesser degree, eastern Asia (Xu et al., 2010). The ceratopsid skull, representing some of the largest among terrestrial vertebrates (Lehman, 1998), is typically characterized by nasal and postorbital horncore outgrowths, and a large, shield-like parietosquamosal frill (Sampson, Ryan & Tanke, 1997). The two ceratopsid subfamilies Centrosaurinae and Chasmosaurinae can be distinguished, in part, by the relative size of the frill, being proportionately longer in the latter (Lehman, 1990).

The Upper Cretaceous (Campanian) Dinosaur Park Formation (DPF) of southern Alberta and Saskatchewan preserves a diverse assemblage of both centrosaurines and chasmosaurines, the latter of which includes *Chasmosaurus belli* (Lambe, 1902), *C. russelli* (Sternberg, 1940), *Vagaceratops irvinensis* (Holmes et al., 2001), *Mercuriceratops gemini* (Ryan et al., 2014), and material possibly referable to *Spiclypeus shipporum* (Mallon et al., 2016).

The vast majority of DPF chasmosaurine material is derived from the Dinosaur Provincial Park (DPP) region of southern Alberta, where this formation is approximately 70 m thick and entirely exposed (Eberth, 2005), and has been intensively sampled for more than 120 years (Lambe, 1902). Chasmosaurine specimens have also been recovered from the more remote and less extensive exposures of the DPF in southeastern Alberta, but this region has received comparatively less attention. This region has nevertheless produced several significant specimens, including the holotypes of *Chasmosaurus russelli* (Sternberg, 1940) and *V. irvinensis* (Holmes et al., 2001), and possible remains of *S. shipporum* (Mallon et al., 2016) and has contributed to a better understanding of the spatiotemporal distribution of chasmosaurines within the DPF.

### Geological setting

The Belly River Group is a predominantly terrestrial sedimentary sequence that was deposited along the western margin of the Western Interior Seaway (Eberth, 2005). This group includes, in ascending stratigraphic order, the Foremost, Oldman, and DPFs. The Oldman and DPFs both represent large-scale alluvial fans that thicken toward their respective sediment sources along the rising Cordillera to the west (Eberth & Hamblin, 1993). The overlap between these wedge-shaped formations results in their contact becoming younger to the south and east. Alluvial and paralic sediments of the DPF represent an overall transgression, transitioning from a sandy to muddy to coaly (Lethbridge Coal Zone; LCZ) intervals, and culminating in marine shale of the overlying Bearpaw Formation.

## Materials and Methods

This study reports on three partial chasmosaurine skulls (CMN 8802, CMN 34829, and TMP 2011.053.0046) collected from the DPF, and age-equivalent sediments of the uppermost Oldman Formation, of southern Alberta. TMP 2011.053.0046 was collected under a Palaeontological Research Permit to DCE (3951–803) in 2011. No permits were required for CMN 8802 and CMN 34829, as they were collected (1937 and 1915, respectively) before the Alberta Historical Resources Act was extended (July 5, 1978) to protect Alberta fossils. Sternberg (1940) originally assigned CMN 8802 to *C. russelli*, but only provided a brief description of this specimen, and did not figure it. Godfrey & Holmes (1995) acknowledged the existence of CMN 34829, and suggested that it might represent *Chasmosaurus*, but could not confirm this taxonomic assignment, given the specimen’s then-unprepared state. A re-description and description of CMN 8802 and CMN 34829, respectively, is warranted, given their relatively well-preserved states, and because CMN 8802 was collected from the uppermost Oldman Formation of southeastern Alberta. TMP 2011.053.0046 has not been previously described, and also warrants description, as it was also collected from the relatively under-sampled Campanian exposures of southeastern Alberta. In this study, the most recent diagnosis of *Chasmosaurus* from Campbell et al. (2016) is used.

Photographs were taken using a Canon E03 Rebel T5i digital SLR camera with an 18–55 mm lens, and Fujifilm Finepix A900 camera, with alterations (i.e., brightness/contrast adjustments, and background removal) performed in Adobe Photoshop CS5.1. Figures were prepared in Adobe Illustrator CS5.1. Measurements were taken to the nearest mm using digital calipers for measurements up to 300 mm, and a cloth measuring tape for those over 300 mm.

## Results

### Redescription of CMN 8802 (*Chasmosaurus russelli* paratype)

**Figure 1:**
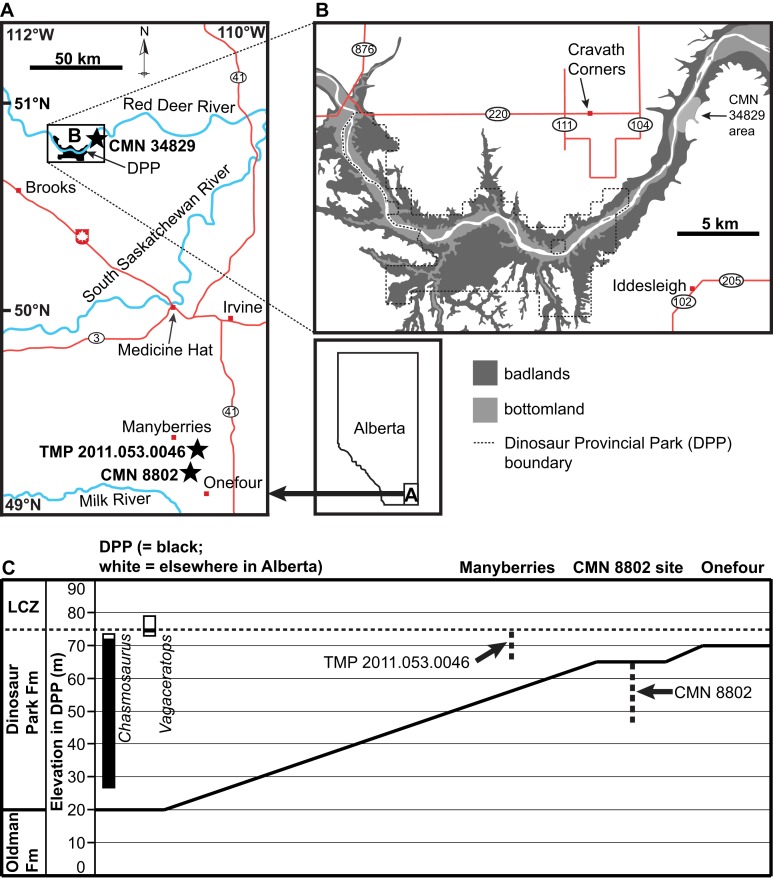
Geographic and stratigraphic positions of the chasmosaurine skulls described in this study (black stars). (A) Geography of southeastern Alberta, showing the locations of CMN 8802 (*Chasmosaurus* sp.), CMN 34829 (*Chasmosaurus* sp.), and TMP 2011.053.0046 (*Vagaceratops* sp.). (B) Geography of Dinosaur Provincial Park (DPP) area, where CMN 34829 was collected. (C) Generalized stratigraphic relationships of the Dinosaur Park and Oldman formations between DPP and Onefour, Alberta, modified from Eberth & Hamblin (1993: Fig. 19a) and Eberth (2005: Fig. 3.1). Stratigraphic positions of CMN 8802 and TMP 2011.053.0046 are shown in relation to the complete, composite stratigraphic ranges of each of *Chasmosaurus* and *Vagaceratops*, modified from Campbell et al. (2016: Fig. 4). CMN 8802 was collected within 20 m of the Dinosaur Park Formation contact, and TMP 2011.053.0046 was collected within 10 m of the base of the Lethbridge Coal Zone (LCZ); the stratigraphic position of CMN 34829 is not known.

CMN 8802 was collected by Sternberg in 1937 south of Manyberries, Alberta (Fig. 1). Sternberg (1940: 478) stated that he had collected the skull “near the same horizon as [CMN] 8800 in the northeast ¼, section 10, township 2, range 5, west of 4th Meridian, or about 13½ miles south and 3¼ miles east of Manyberries” (the *C. russelli* holotype, CMN 8800, was found just below the LCZ, for reference (Campbell et al., 2016)). This description is contradictory, as the given quarter section is situated approximately 10 km farther south than the given distance from Manyberries. However, according to the CMN collections database, CMN 8802 is listed as having been collected at site “P-3708, Legal subdivision 14, in northwest ¼ of section 10, township 3, range 5, west of 4th Meridian, on south side of west branch of creek that runs past Joe Gilchrist’s Ranch House, approximately 10 feet below prairie (marker # 118 in quarry)” (M. Currie, personal communication, 2014). This latter quarter section matches the distance from Manyberries given in the former description. A quarry stake labelled as GSC 118 (Geological Survey of Canada) was located by DCE in 2016 in the close vicinity of the latter locality; precise locality information on file at the CMN. This stake lies in the uppermost sediments of the Oldman Formation, immediately below the contact with the DPF, which itself is approximately 10 m below the base of the LCZ (Fig. 1). The DPF contact is not present in the immediate area of the stake, so a conservative estimate is that the stake lies within 20 m of the DPF contact. The stake horizon is age-equivalent to the upper DPF, as exposed in DPP.

CMN 8802 was briefly described (but not figured) and assigned by Sternberg (1940) as a paratype of *C. russelli*. In September of 1941, it was severely damaged while in transit, and reported as having been destroyed, except for the lower jaws (Godfrey & Holmes, 1995). However, this specimen was rediscovered in the CMN collections by one of the authors (MJR) in 2011 as a more intact skull that had been renumbered as CMN 8798—the same number as a *Centrosaurus apertus* skull (Brown, Russell & Ryan, 2009). In 1975, Wann Langston Jr., then curator at the NMC (National Museums of Canada; now CMN), made brief reference to the innervation pattern of a prepared *Chasmosaurus* braincase (Langston, 1975); this specimen undoubtedly represents CMN 8802, as no other *Chasmosaurus* skull at the NMC would have fit this description.

CMN 8802 consists of the rostral, premaxillae, postorbitals, jugal-quadratojugal-quadrate complexes, braincase, parietosquamosal frill, isolated epiossifications, and lower jaws (Figs. 2–9). Additional small cranial fragments are non-diagnostic and cannot be reattached.

**Figure 2:**
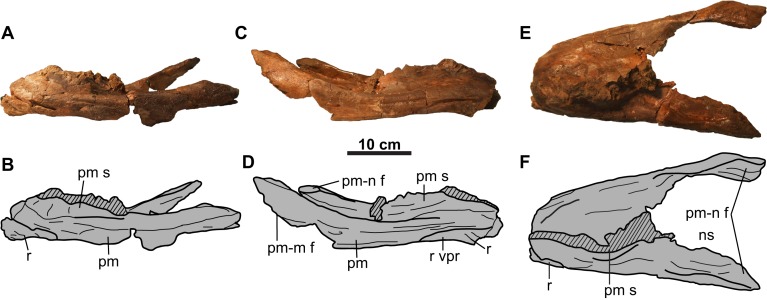
Rostral and premaxillae of CMN 8802 (*Chasmosaurus* sp.). Elements in (A–B) left lateral, (C–D) right lateral, and (E–F) dorsal views. Hashed areas represent broken bone. For abbreviations, see Anatomical Abbreviations.

**Figure 3:**
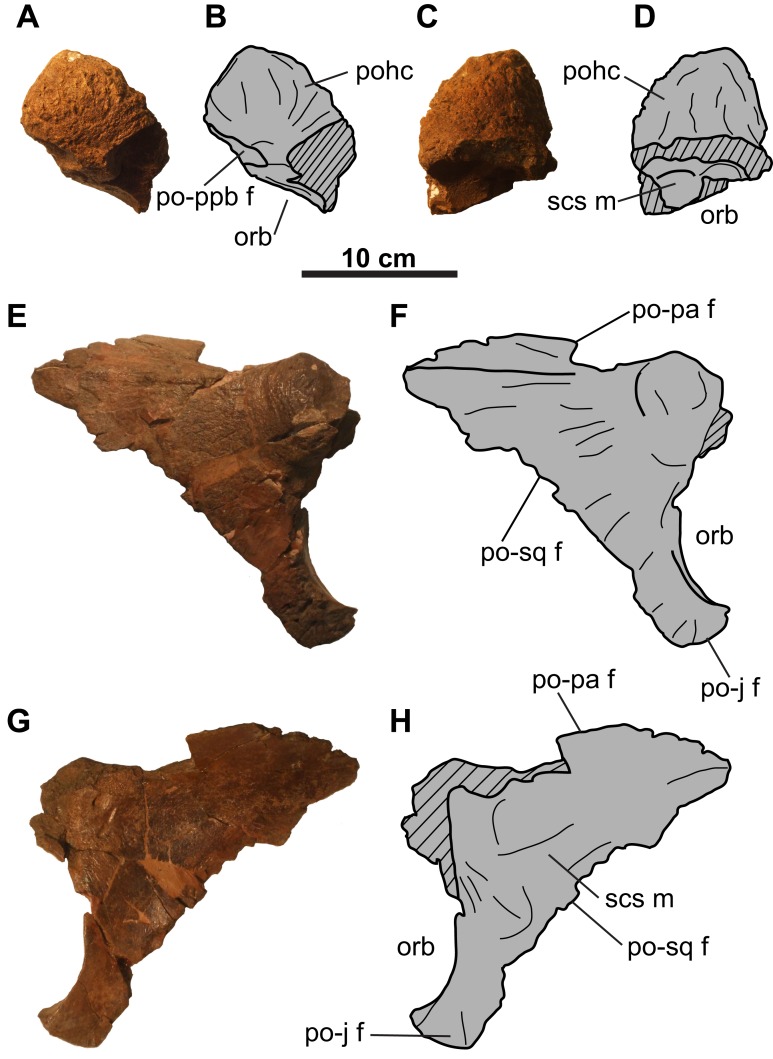
Postorbitals of CMN 8802 (*Chasmosaurus* sp.). Left postorbital in (A–B) lateral and (C–D) medial views. Right postorbital in (E–F) lateral and (G–H) medial views. Hashed areas represent broken bone. For abbreviations, see Anatomical Abbreviations.

**Figure 4:**
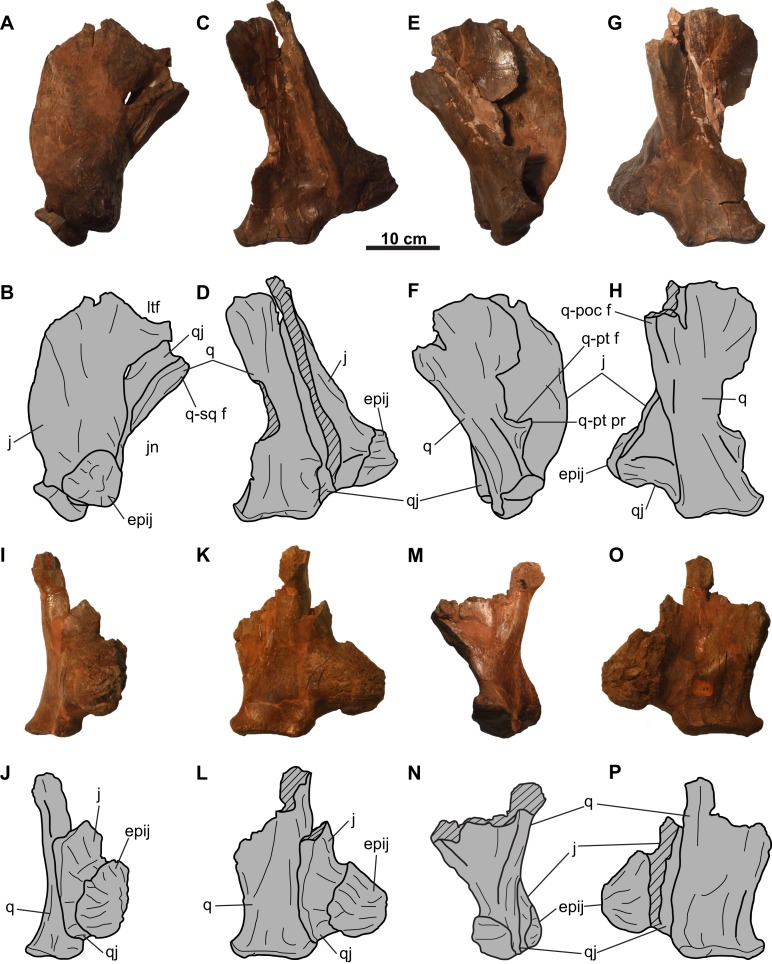
Jugal, quadratojugal, and quadrate complexes of CMN 8802 (*Chasmosaurus* sp.). Left complex in (A–B) lateral, (C–D) anterior, (E–F) medial, and (G–H) posterior views. Right complex in (I–J) lateral, (K–L) anterior, (M–N) medial, and (O–P) posterior views. Hashed areas represent broken bone. For abbreviations, see Anatomical Abbreviations.

**Figure 5:**
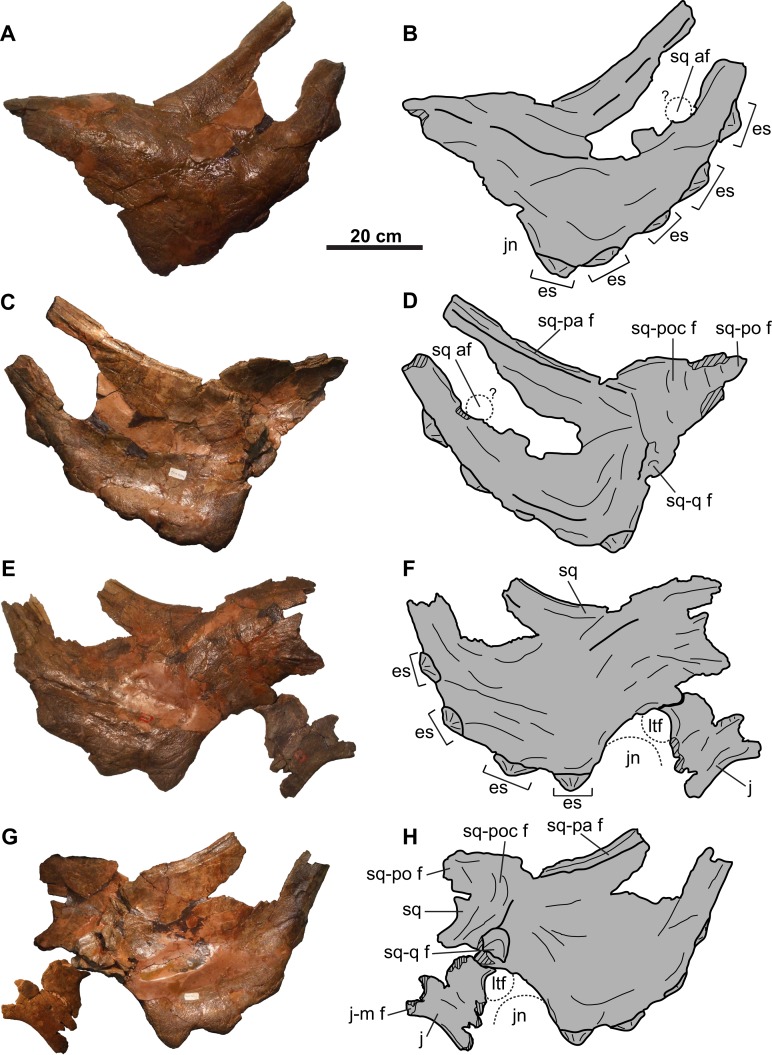
Squamosals of CMN 8802 (*Chasmosaurus* sp.). Left squamosal in (A–B) dorsal, and (C–D) ventral views. Right squamosal and jugal in (E–F) dorsal, and (G–H) ventral views. Dotted line in (B) and (D) represents inferred margin of accessory squamosal fenestra. Dotted lines in (F) and (H) represent inferred margins of lateral temporal fenestra and jugal notch. Hashed areas represent broken bone. For abbreviations, see Anatomical Abbreviations.

**Figure 6:**
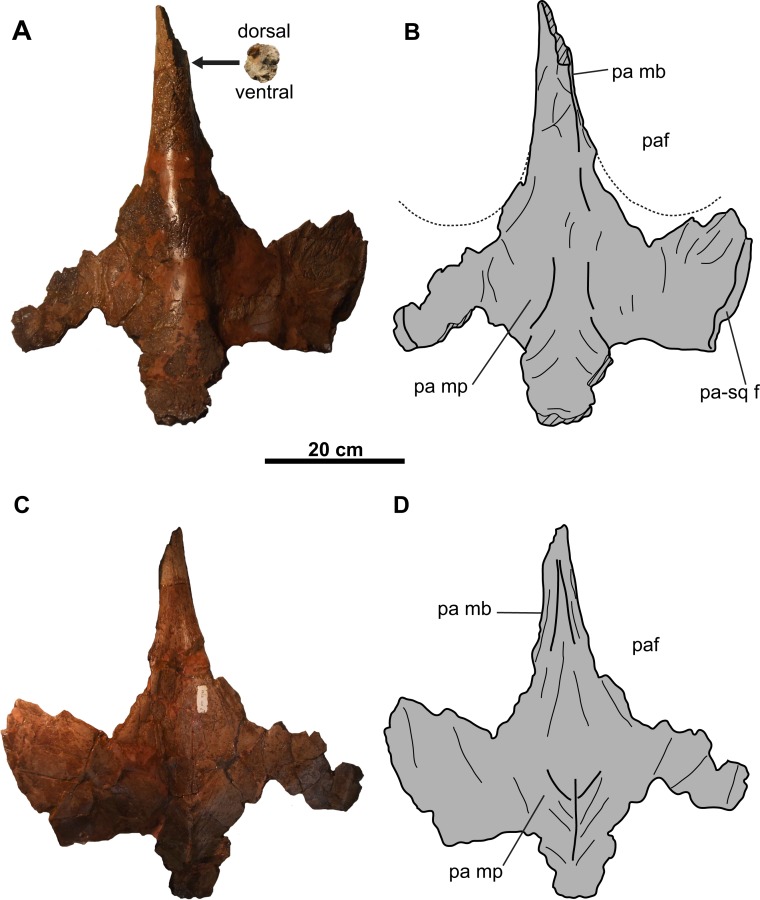
Parietal of CMN 8802 (*Chasmosaurus* sp.). Parietal in (A–B) dorsal and (C–D) ventral views. Dotted lines in (B) represent inferred margins of parietal fenestrae. Hashed areas represent broken bone. For abbreviations, see Anatomical Abbreviations.

**Figure 7:**
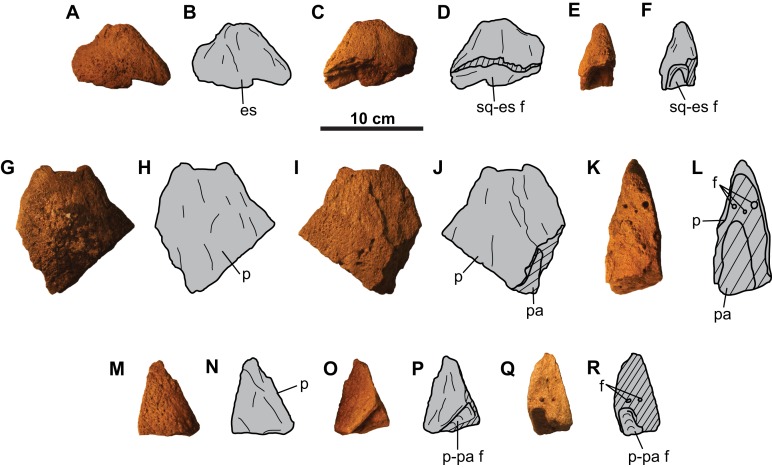
Frillepiossifications of CMN 8802 (*Chasmosaurus* sp.). Probable episquamosal (A–F) in (A–B) dorsal (?), (C–D) ventral (?), and (E–F) cross-sectional views. Probable epiparietals: first epiparietal (G–L) in (G–H) dorsal (?), (I–J) ventral (?), and (K–L) cross-sectional views; and second epiparietal (M–R) in (M–N) dorsal (?), (O–P) ventral (?), and (Q–R) cross-sectional views. Hashed areas represent broken bone. For abbreviations, see Anatomical Abbreviations.

**Figure 8:**
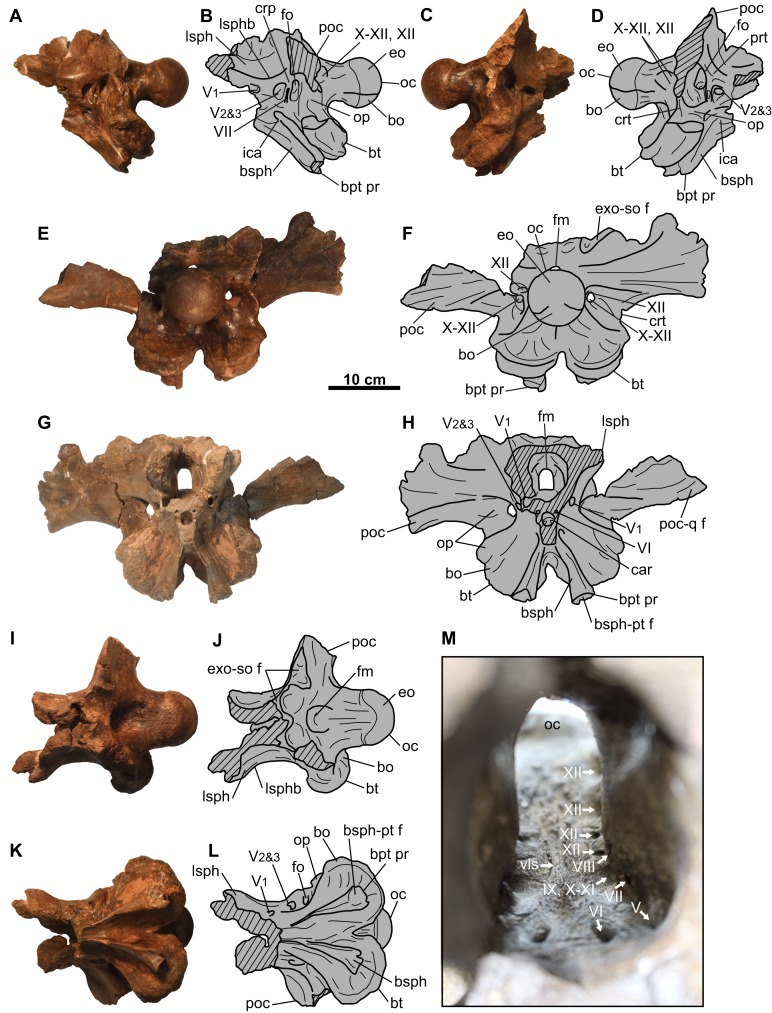
Braincase of CMN 8802 (*Chasmosaurus* sp.). Braincase in (A–B) left lateral, (C–D) right lateral, (E–F) posterior, (G–H) anterior, (I–J) dorsal, and (K–L) ventral views. Endocranium in (M) anterior view. The paroccipital process fragments are not shown in (A–D) and (I–L). Hashed areas represent broken bone. For abbreviations, see Anatomical Abbreviations.

**Figure 9:**
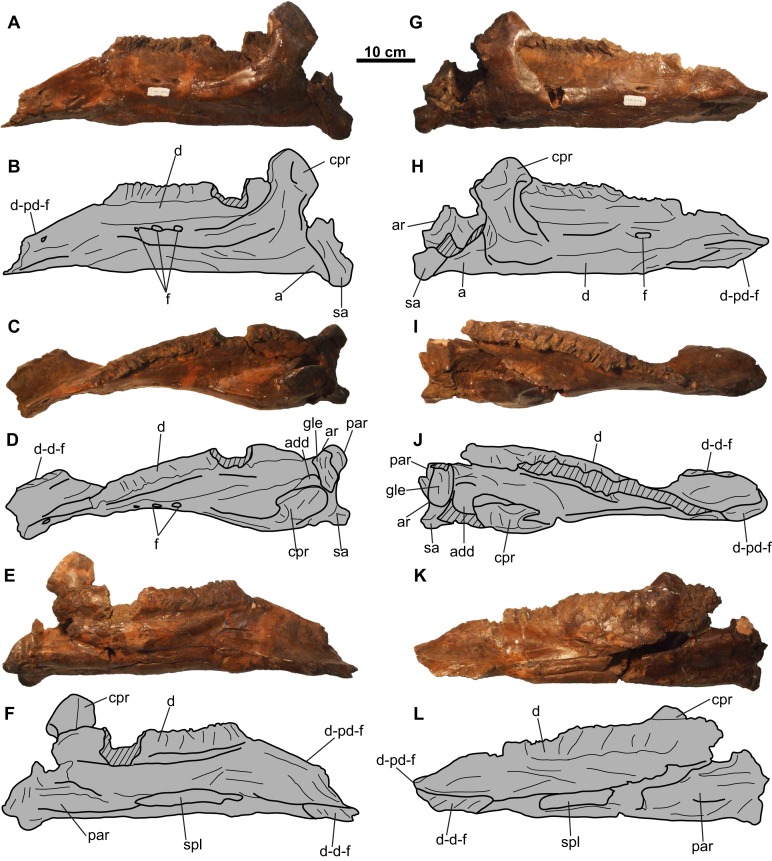
Lower jaws of CMN 8802 (*Chasmosaurus* sp.). Left jaw in (A–B) lateral, (C–D) dorsal, and (E–F) medial views. Right jaw in (G–H) lateral, (I–J) dorsal, and (K–L) medial views. Hashed areas represent broken bone. For abbreviations, see Anatomical Abbreviations.

### Snout region

**Rostral**: Only the diagnostic, elongate chasmosaurine (Sampson et al., 2010) ventral processes of the rostral are preserved (Fig. 2). These processes remain in articulation with the anteroventral surface of the premaxillae, although the sutures are open externally.

**Premaxilla**: Both premaxillae are preserved and mostly complete (Fig. 2). Like other chasmosaurines, they are anteroposteriorly elongate, have a horizontal ventral (oral) margin, and lack a ventral recess at the base of the premaxillary septum (Lehman, 1990). The posterior processes (Figs. 2C and 2D) bear facets for the non-preserved maxillae and nasals.

### Circumorbital and cheek regions

**Postorbital**: The left postorbital is represented by a short (∼71 mm as measured from the orbital margin) horncore (Figs. 3A–3D). It has a rounded apex, and a vascularized, rugose surface. The anteroventral margin bears a facet to receive the non-preserved palpebral. Medial and ventral to the horncore is the supracranial sinus (Figs. 3C and 3D).

The more complete right postorbital preserves the posterior orbital margin (Figs. 3E–3H). Although incomplete, the height of the orbit is approximately 100 mm. Anterodorsally, the bone thickens to form the posterior half of the horncore. Ventral and posterior to the orbital margin are the articulating facets for the jugal and squamosal. The posteromedial margin of the postorbital preserves the contact for the parietal. The smooth, gently concave medial surface forms the lateral margin of the supracranial sinus (Figs. 3G and 3H).

**Jugal**: Both jugals are partly represented, with the right element better preserved (Figs. 4 and 5). The anterior and orbital margins of each jugal are not preserved. The smooth medial surface of the jugal preserves a rugose facet for the maxilla (Figs. 5G and 5H; sensu Maidment & Barrett (2011: Fig. 4D)). The jugal forms an anteroposteriorly-wide wing that projects and tapers posteroventrally. The wing articulates medially with the quadratojugal, and at its tip, with a large (66 mm tall; comparable in size with the *Chasmosaurus* sp. skull CMN 8801, but smaller than *Pentaceratops*; Lehman, 1998) trihedral epijugal (Fig. 4).

The jugal articulates with the squamosal posteriorly, together forming the anterior and posterior margins of the lateral temporal fenestra, respectively (Figs. 5E–5H). This fenestra is incompletely preserved, but has a maximum preserved diameter of approximately 73 mm. The left jugal bears a prominent, posteriorly projecting process, which forms the anteroventral margin of the lateral temporal fenestra (Figs. 4A and 4B). This process is broken posteriorly, and it cannot be determined whether it would have contacted the squamosal, or if the underlying quadratojugal contributed to the margin of the lateral temporal fenestra. Ventral to the lateral temporal fenestra is the wide jugal notch (Fig. 5).

**Quadratojugal**: Portions of both quadratojugals are preserved (Fig. 4). Only a limited description of this element can be made, as much of its morphology is obscured by the jugal laterally and quadrate medially. Dorsally, the quadratojugal is mediolaterally compressed, but thickens ventrally (Fig. 4). The ventromedial surface of the element has a cup-like socket to receive the ventrolateral end of the quadrate (Figs. 4A–4D and 4I–4L). The posteroventral end of the quadratojugal articulates with the ventral edge of the epijugal (Figs. 4G, 4H, 4O and 4P).

**Quadrate**: Both quadrates are represented, with the left element better preserved (Fig. 4). Its ventral end is 108 mm wide and bilobate (Fig. 4) and would have articulated with the glenoid fossa on the lower jaw (Fig. 9). More dorsally, the pterygoid process projects from the medial margin (Figs. 4E and 4F). Dorsal to this process is the facet for the pterygoid, which is bordered by a ridge that lines the medial margin of the quadrate and extends onto the process.

### Parietosquamosal frill

**Squamosal**: Both elements are represented, but approximately one-third of the posterior-most portion of each element is missing (Fig. 5). They are anteroposteriorly elongate, as in other chasmosaurines (Lehman, 1990). The more complete right squamosal has a maximum width (as measured from the lateral margin of the anterior-most episquamosal to the squamosal medial margin) of 436 mm.

The fluted sutural surface for the postorbital (Figs. 5C, 5D, 5G and 5H) is present on the anteromedial squamosal surface. The squamosal would originally have had an infratemporal process that formed the posteroventral margin of the lateral temporal fenestra, as in other ceratopsids (e.g., *Chasmosaurus* [CMN 2280], Campbell et al., 2016; *Pachyrhinosaurus* [TMP 1987.055.0261], Currie, Langston & Tanke, 2008; *Triceratops* [YPM 1822], Forster, 1996), but it is broken off. Above this fenestra, on the medial surface, lies the facet for the quadrate, and above that, the facet for the paroccipital process (Figs. 5C, 5D, 5G and 5H). The medial margin of the squamosal forms the lateral margin of the dorsal temporal fenestra.

Further posteriorly along the ventromedial margin lies the anteroposteriorly elongate facet for the lateral parietal bar (Figs. 5C, 5D, 5G and 5H). The more complete facet on the left squamosal (Figs. 5C and 5D) is concave anteriorly, but gradually flattens and disappears posteriorly. It cannot be determined if the squamosal formed part of the lateral margin of the parietal fenestra, or if the lateral bar excluded it. On the dorsal surface, there is a thickened, rounded swelling along the medial margin, as in most other chasmosaurines (excluding e.g., *Kosmoceratops*, *Ojoceratops*, *Nedoceratops*, and *Triceratops*) but not in centrosaurines (Sampson et al., 2010).

The lateral margin of an accessory fenestra (Figs. 5A–5D) is present on the left squamosal, situated medial to the posterior-most episquamosal. The preserved fenestra margin is smooth and not swollen, with vascular grooves continuing right up to the edge of the fenestra.

The left squamosal (Figs. 5A–5D) preserves five approximately equal-sized (between 72 and 99 mm long), triangular episquamosals, while the right (Figs. 5E–5H) preserves four. The total episquamosal count cannot be determined due to incomplete preservation. Coossification between the squamosal and episquamosals becomes less complete in an anteroposterior direction, indicating an anteroposterior direction of fusion, as in other chasmosaurines (Campbell et al., 2016); in contrast, centrosaurine episquamosals fuse in the opposite direction (Sampson, Ryan & Tanke, 1997).

**Parietal**: The parietal (Fig. 6) is represented by most of the median bar and the anterior median platform. The highly vascularized dorsal surface (Figs. 6A and 6B) is convex along the midline, while the relatively smooth ventral surface (Figs. 6C and 6D) is concave along the midline, but gradually flattens posteriorly. Portions of the facets for the squamosals are preserved on its dorsolateral margins (Figs. 6A and 6B). Posteriorly, the platform transitions into the median bar, which is narrow and strap-like, as in *Chasmosaurus*, *Vagaceratops*, *Agujaceratops*, *Utahceratops*, and *Pentaceratops* (Sampson et al., 2010). The posterior-most preserved end of the median bar is rectangular and slightly transversely compressed in cross-section (57 mm tall and 53 mm wide). Although incomplete, the parietal fenestrae would have been anteroposteriorly elongate, as in most other chasmosaurines.

**Epiossifications**: Three additional parietosquamosal frill epiossifications are preserved. The first one is short (approximately 60 mm tall), has a distinct, unmodified base, and does not appear to have been coossified with the underlying frill (Figs. 7A–7F). The triangular shape and size of this element is consistent with the articulated episquamosals (Figs. 5A–5H), and is tentatively interpreted as a posterior, unfused episquamosal that became detached post-mortem.

The second epiossification (Figs. 7G–7L) is large (about 130 mm tall) and straight with gently convex dorsal and ventral surfaces. It is broken, revealing information on its relationship with the underlying frill bone (Figs. 7I–7L). The epiossification overlaps (at least 60 mm, proximodistally) the dorsal and ventral surfaces of the underlying bone, forming a parabolic coossified sutural contact. There are some vascular canals visible in cross-sectional view (Figs. 7K and 7L). This epiossification is considerably taller than the episquamosals in Figs. 5 and 7A–7F (maximum height approximately 60 mm), and its advanced degree of fusion with the underlying bone precludes it from being a posterior episquamosal, as such episquamosals were likely unfused. Accordingly, we interpret this element as an epiparietal (EP).

The third, incomplete epiossification (Figs. 7M–7R) has a preserved length and height of approximately 60 and 75 mm, respectively. The overall shape and thickness of this element suggests that it may have been as large as the putative EP; therefore, we tentatively interpret this element as an unfused EP.

### Braincase

The braincase (Fig. 8) is disarticulated from the rest of the skull. It is almost complete, missing only the anterior portion, supraoccipital, and the distal ends of the paroccipital processes. The supraoccipital does not appear to be fused to the underlying exoccipitals, but otherwise all elements are coossified, as in osteologically mature ceratopsids (Godfrey & Holmes, 1995). External sutures are obliterated except for part of the suture between the basioccipital and exoccipitals, which can be faintly discerned on the occipital condyle and condylar neck (Figs. 8A–8F). The general extent of most cranial elements in CMN 8802 cannot be determined, and are inferred based on those of other ceratopsids (see below). Nerve and artery canals can be traced through the hollow endocranium using a stiff but flexible brush bristle.

The occipital condyle has a circular outline in posterior view, measuring 76 mm horizontally across (Figs. 8E and 8F). The basioccipital forms the ventral third of the condyle, and the paired exoccipitals form the dorsal two-thirds. The condylar neck has a uniform width in dorsal view (Figs. 8I and 8J), but it narrows anteriorly in lateral view (Figs. 8A–8D).

The foramen magnum lies above the base of the condylar neck, and is taller (30 mm) than wide (18 mm) (Figs. 8E, 8F, 8I and 8J). The foramen is bordered on all sides by the paired exoccipitals, as in relatively mature individuals, and unlike the immature condition in which the supraoccipital forms the dorsal margin of the foramen (e.g., *Chasmosaurus* [UALVP 52613], Currie et al., 2016; *Triceratops* [UCMP 154452], Goodwin et al., 2006). The foramen magnum leads anteriorly to the mostly complete endocranial cavity (Figs. 8E–8H and 8M). The floor of the endocranium has a prominent median canal for the ventral longitudinal sinus (Fig. 8M; Witmer & Ridgely, 2008). Lateral to this canal is an anteroposteriorly-arranged series of foramina (described below). The facet for the supraoccipital is visible on the dorsal surface of the exoccipitals (Figs. 8E, 8F, 8I and 8J). Posterior to this facet, there is a prominent median ridge separating two fossae.

The basal tubera flare out ventrolaterally below the condylar neck (Fig. 8). In ventral view, the region just anterior of the basal tubera where the basilar artery would be expected (Maidment & Barrett, 2011) is obscured by plaster (Figs. 8K and 8L). Each tuber is formed mostly by the basioccipital, except for the anterolateral portion, which is formed by the ventral end of the opisthotic (Forster, 1990); in anterior view, there is a distinct notch on the lateral margin of the tuber, which represents the border between the basioccipital ventrally and the opisthotic dorsally (Figs. 8G and 8H). The foramen for the exit (maximum diameter [MD] = 7.3 mm) of the posterior-most canal of the hypoglossal (cranial nerve XII) nerve is situated above the tubera, laterally adjacent to the condylar neck (Figs. 8A–8F). This canal enters the floor of the endocranium via the posterior-most foramen (MD = 6.1 mm) in Fig. 8M.

Langston (1975) described three hypoglossal nerve canals, but we observe four. Anteroventral to the exit of the posterior-most canal for the hypoglossal nerve described above, the braincase is pierced by the shared exit (MD = 10.1 mm) for the vagus (cranial nerve X), accessory (cranial nerve XI), and three additional canals of the hypoglossal nerve (Figs. 8A–8F). Internally, the shared canal for cranial nerves X–XII splits and enters the endocranial floor via four foramina situated anterior to the opening for the posterior-most canal of cranial nerve XII (Fig. 8M). The anterior-most and largest of these four foramina (MD = 23.5 mm) bifurcates laterally into two canals—one leading posteriorly to the exit for cranial nerves X–XII, having transmitted cranial nerves X and XI together, and the other leading to the fenestra ovalis, having transmitted the glossopharyngeal nerve (cranial nerve IX; Witmer & Ridgely, 2008). The three more remaining foramina (MDs = 0.6, 2.0, and 2.8 mm, moving posteriorly; Fig. 8M) converge at the exit for cranial nerves X–XII. Three cranial nerve XII canals, with the anterior two joining the shared canal for cranial nerves X–XI, have been previously reported for *Triceratops* (Forster, 1996), as well as *Pachyrhinosaurus canadensis* (Langston, 1975) and *P. lakustai* (Currie, Langston & Tanke, 2008), while only two have been reported for *Centrosaurus* (Witmer & Ridgely, 2008).

The dorsoventrally tall and anteroposteriorly compressed paroccipital processes (Figs. 8E–8H) are each formed by the fusion of the exoccipital posteriorly, and the opisthotic anteriorly and distally (Forster, 1996). The distal-most ends of these processes are not preserved, but would have articulated with the anteroventral surface of the squamosals (Figs. 5C, 5D, 5G and 5H). The dorsal margins of the processes are poorly preserved, but would have contacted the anteroventral surface of the parietal (Godfrey & Holmes, 1995). Part of the facet for the posterodorsal end of the quadrate is visible on the left anteroventral surface of the left paroccipital (Figs. 8G and 8H). Ventrally, a ridge (crista tuberalis; Witmer & Ridgely, 2008) continues from the base of the paroccipital process and onto the basal tuber (Figs. 8C–8F).

The basipterygoid processes lie anterior to the basal tubera (Figs. 5A–5D). Each process is formed by the basisphenoid, and originates dorsally as a rounded, thickened ridge, which descends and projects posteroventrally and laterally from the braincase (Figs. 8A–8H, 8K and 8L). The incompletely preserved distal end of each process has a well-defined facet for the non-preserved pterygoid (Godfrey & Holmes, 1995).

The fenestra ovalis (MD = 14.8 mm) is situated anterior to the base of the paroccipital process (Figs. 8A–8D, 8K and 8L), and would have transmitted cranial nerve IX (Witmer & Ridgely, 2008). In ceratopsids, the anterior margin of the fenestra ovalis forms the border between the opisthotic posteriorly and the prootic anteriorly (Lehman, 1989). Moving internally, the cranial nerve IX canal merges with the anterior-most branch of the shared canal for cranial nerves X–XI, and enters the endocranium via a large opening (Fig. 8M). Anterodorsal to this large, endocranial opening is a small foramen (MD = 2.8 mm; Fig. 8M), which also exits the braincase via the fenestra ovalis. This foramen is interpreted herein as being for the vestibulocochlear nerve (cranial nerve VIII), which enters the inner ear as both the vestibular and cochlear branches, and exits the inner ear via the fenestra ovalis (Witmer & Ridgely, 2008).

Anteroventral to the endocranial opening for cranial nerve VIII is the opening for the facial nerve (MD = 3.6 mm; cranial nerve VII; Fig. 8M; Witmer & Ridgely, 2008). Cranial nerve VII exits the braincase through a dorsoventrally elongate opening (MD = 5.5 mm) situated immediately anterior to the fenestra ovalis (Figs. 8A and 8B). Anterodorsal to the exit for cranial nerve VII is a ridge (crista prootica) that continues posteriorly across the dorsal margin of the fenestra ovalis and onto the paroccipital process (Figs. 8A–8D).

Anterior to the cranial nerve VII exit lies the foramen (MD = 27.3 mm) through which the maxillary and mandibular branches (cranial nerve V_2&3_) of the trigeminal nerve exit the braincase (Figs. 8A–8D, 8K and 8L). Anterior to this foramen is the exit (MD = 9.3 mm) for the ophthalmic branch (cranial nerve V1) of this nerve (Figs. 8A, 8B, 8K and 8L). The border between the prootic posteriorly and the laterosphenoid anteriorly probably lies between the cranial nerve V_1_ and V_2&3_ exits (Lehman, 1989) although no suture is visible. Anterodorsal to these exits is the anterodorsally inclined laterosphenoid buttress (Figs. 8A–8D, 8I and 8J). The canals for the cranial nerve V_1_ and V_2&3_ branches enter the endocranium via a single, large foramen (MD = 11.7 mm; Figs. 8G, 8H and 8M).

In lateral view, the anterodorsally inclined internal carotid artery canal enters the braincase directly below the cranial nerve V_2&3_ exit (Figs. 8A–8D). The left and right carotid foramina (MD = 8.5 mm) converge anteriorly into a single, large foramen (MD = 18.5 mm; cerebral carotid artery canal) (Figs. 8G and 8H). Anteriorly, the cerebral carotid artery canal would have led into the non-preserved pituitary (hypophyseal) fossa of the endocranium (Witmer & Ridgely, 2008). In anterior view (Figs. 8G and 8H), there are two small foramina (MD = 4.6 mm) situated dorsolaterally to the cerebral carotid artery canal. These two foramina would have each transmitted the abducens nerve (cranial nerve VI), which can be traced posterodorsally into the endocranium (MD = 5.5 mm; Fig. 8M).

### Lower jaws

Both lower jaws are nearly complete (approximately 630 mm long), missing only the predentaries (Fig. 9). Several of the sutures between the elements of the lower jaws are obscured with plaster.

**Dentary**: The toothrow is incomplete and weathered, making a tooth count impossible (Fig. 9). The coronoid process obscures the posterior end of the tooth row in lateral view (Fig. 9). The coronoid process is not as laterally displaced from the rest of the lower jaw to the degree seen in *Arrhinoceratops* ([CMN 8882, ROM 1439] Mallon et al., 2014; Mallon, Ryan & Campbell, 2015), but is more consistent with that of other chasmosaurines (e.g., *Chasmosaurus* [ROM 843]).

The ventral margin of each dentary is relatively straight, as in some specimens of *Chasmosaurus* (e.g., CMN 2280, CMN 8801) and *Vagaceratops* (CMN 41357). Convex ventral margins also occur in some *Chasmosaurus* specimens (e.g., CMN 1254, ROM 843). The shape of the ventral margin does not appear to be ontogenetically variable within *Chasmosaurus*, as convex margins are present in both small (i.e., CMN 1254) and large (i.e., ROM 843) skulls.

**Splenial**: The splenial is narrow and mediolaterally compressed, and tapers at each end. It is nested within an anteroposteriorly-oriented groove on the ventromedial surface of the dentary (Figs. 9E, 9F, 9K and 9L). The splenial extends the length of the tooth row.

**Angular**: The angular is visible in lateral view and articulates with the dentary and surangular immediately below the base of the coronoid process (Figs. 9A, 9B, 9G and 9H). It has a flat ventral margin, confluent with that of the dentary, except for its posterior-most end, where it curves ventrally before contacting the surangular.

**Surangular**: The surangular is visible in lateral view, situated posterodorsal to the angular, and extends further ventrally than the latter. It forms most of the lateral margin of the adductor fossa, as well as a small portion of the glenoid fossa (Figs. 9A–9D and 9G–9J).

**Articular**: The articular is located medial to the surangular (Figs. 9C, 9D and 9G–9J) and forms a concave dorsal surface for the ventral end of the quadrate (Fig. 4). It forms the medial wall and most of the floor of the adductor fossa, as well as most of the glenoid fossa.

**Prearticular**: The prearticular (Figs. 9C–9F and 9I–9L) is visible medially, and is situated below the coronoid process and articular. It extends anterior to the coronoid process, although its anterior-most extent cannot be determined.

### Description of CMN 34829

**Figure 10:**
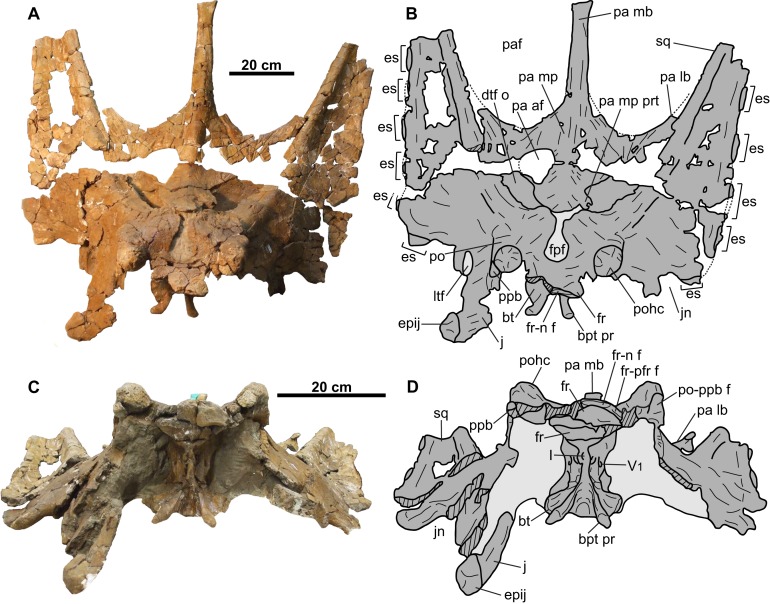
Skull of CMN 34829 (*Chasmosaurus* sp.). Skull in (A–B) dorsal and (C–D) anterior views. Dotted lines in (B) represent inferred margins of missing bone. Hashed areas represent broken bone. Light grey indicates rock matrix. For abbreviations, see Anatomical Abbreviations.

**Figure 11:**
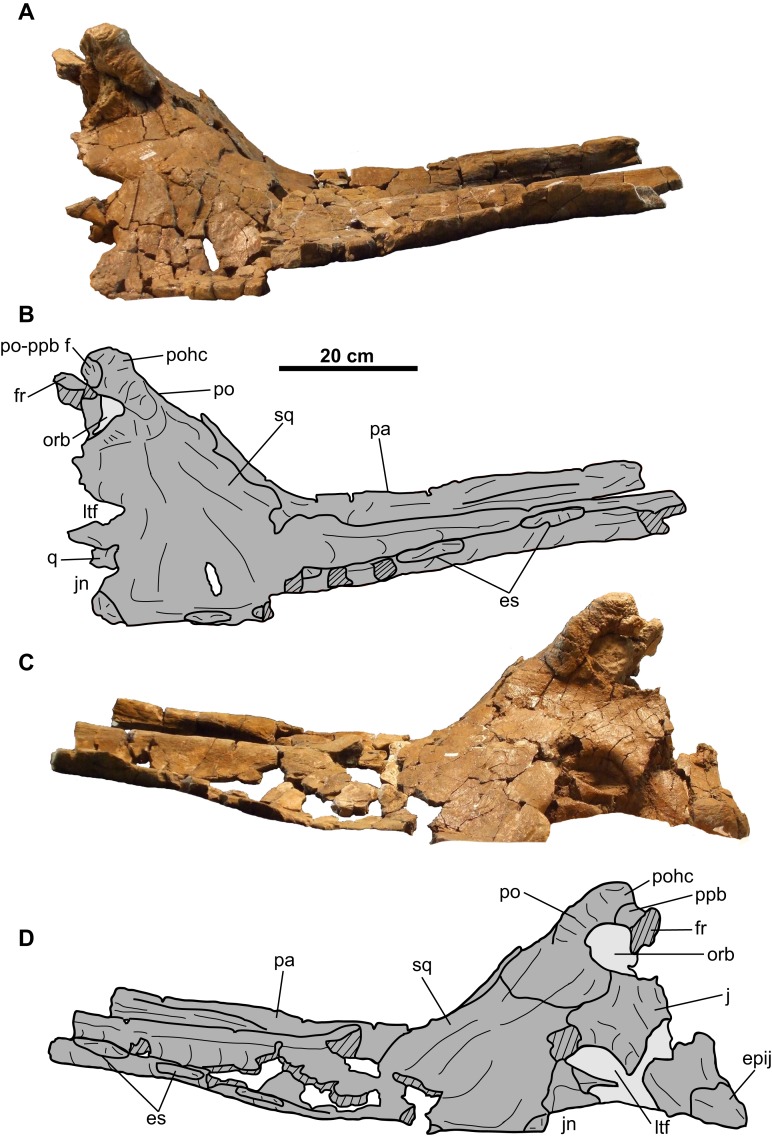
Skull of CMN 34829 (*Chasmosaurus* sp.). Skull in (A–B) left lateral and (C–D) right lateral views. Hashed areas represent broken bone. Light grey indicates rock matrix. For abbreviations, see Anatomical Abbreviations.

**Figure 12:**
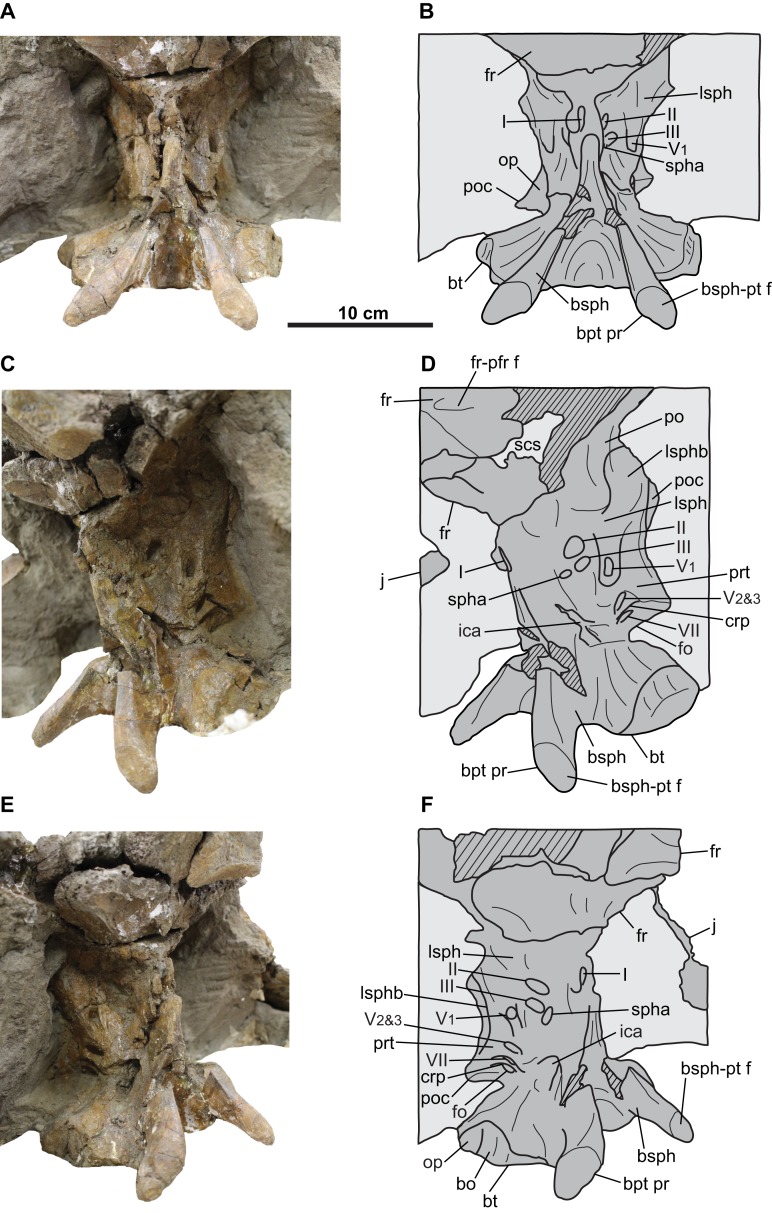
Braincase of CMN 34829 (*Chasmosaurus* sp.). Braincase in (A–B) anterior, (C–D) left anterolateral, and (E–F) right anterolateral views. Hashed areas represent broken bone. Light grey indicates rock matrix. For abbreviations, see Anatomical Abbreviations.

CMN 34829 (Figs. 10–12) is a highly fractured, partial skull that was collected by Sternberg (1915), from CMN fossil site P-1532. The description for this locality is as follows: “near lower end of Deadlodge Canyon, on the east side of the Red Deer River, almost east of Cravath Corners Post Office”. Cravath Corners was a small settlement that existed from 1910 to 1926, located on what is now Township Road 220, between Range roads 111 and 104, north of DPP (Fig. 1). Due east of this settlement, the Red Deer River trends roughly north-south, with the east bank consisting of steep, stratigraphically extensive exposures of the DPF (D. Tanke, 2018, personal communication). This general area is situated approximately 7 km downstream of the present-day borders of DPP (Fig. 1). When discovered by Sternberg (1915), the skull was “in a slide and the crest (i.e., parietosquamosal frill) (was) in pieces.”

The skull includes the skull roof and braincase, most of the circumorbital regions, and most of the parietosquamosal frill (Figs. 10–12). Other small cranial fragments are also associated with this specimen, but they cannot be reattached and appear to be non-diagnostic.

CMN 34829 was noted by Godfrey & Holmes (1995) as being in an unprepared state, but has recently (2011–2012) undergone preparation at the CMN, and is now available for a complete description. Of note, CMN 34829 was misidentified as field number 11, rather than the correct field number 10, in Sternberg (1915). Godfrey & Holmes (1995) also mention another skull (described as field number 10, but actually 11) collected during this field season, but the authors conclude that it was likely destroyed in a fire in the CMN collection facility in the 1930s.

### Circumorbital region

**Postorbital**: Both postorbitals are preserved (Figs. 10 and 11). The sutures for the palpebral and the jugal are visible on the right element (Figs. 11C and 11D). The suture for the squamosal is difficult to discern, due to extensive fracturing of the skull surface. The postorbitals form most of the lateral margins of the frontoparietal fontanelle (Figs. 10A and 10B), as in other chasmosaurines (Lehman, 1990).

The frontoparietal fontanelle is transversely expanded (59 mm wide; Figs. 10A and 10B), as in some taxa (e.g., *Chasmosaurus*, *Vagaceratops*, and *Agujaceratops*), but unlike the reduced (e.g., *Anchiceratops*) or completely roofed over (e.g., *Arrhinoceratops*, *Kosmoceratops*, *Triceratops prorsus*, and some specimens of *Torosaurus latus*; Farke, 2010) conditions in other taxa. More specifically, the fontanelle of CMN 34829 is keyhole shaped, as in *Chasmosaurus* (e.g., AMNH 5402, ROM 843) and *Vagaceratops* (e.g., TMP 1987.045.0001). The frontoparietal fontanelle leads ventrally into the supracranial sinus, which is mostly infilled with matrix.

The postorbital horncores of CMN 34829 (Figs. 10 and 11) are positioned anterior to the center of the orbit. They are short (59 mm) and oriented dorsally. The apices are rounded and pitted, suggesting that the horncores were remodeled down from their original, presumably longer and more pointed condition prior to death.

**Palpebral**: The dorsal portion of the right palpebral is preserved (Figs. 10C, 10D, 11C and 11D). The suture between the palpebral and postorbital is situated immediately ventral to the postorbital horncore. The palpebral has a thickened, rugose surface.

**Frontal**: Both coossified frontals are represented (Figs. 10–12). They form the anterior margin of the frontoparietal fontanelle posteriorly, and articulate with the postorbitals laterally, although these sutures cannot be discerned. The facet for the non-preserved nasal is present on the anterior margin of the left frontal. The lateral margin of the left frontal bears a facet for the non-preserved prefrontal. The frontals are dorsoventrally thick, contacting the laterosphenoids ventrally (Fig. 12). They are deeply excavated posteriorly by the supracranial sinus (see Forster, 1996), part of which can be seen in ventrolateral view (Figs. 12C and 12D).

**Jugal**: The right jugal (Figs. 10, 11C and 11D) is partially preserved and resembles those of other ceratopsids, but is crushed and laterally displaced. The posterodorsal margin is sinusoidal in lateral view, where it contacts the postorbital anteriorly and the squamosal posteriorly. The original shape of the lateral temporal fenestra cannot be determined. A large (47 mm tall), trihedral epijugal is articulated with the jugal (Figs. 11C and 11D).

**Quadrate**: Only the dorsal end of the left quadrate is preserved, however, due to poor preservation, morphological description is impossible (Figs. 11A and 11B).

### Parietosquamosal frill

**Squamosal**: Both squamosals are mostly preserved, except for the posterior-most ends (Figs. 10 and 11). The squamosal is long and triangular as in other chasmosaurines (e.g., *Chasmosaurus* [NHMUK R4948, ROM 843]; *Pentaceratops* [AMNH 1624], Lehman, 1998), and has an estimated total length of 850 mm, which is within the range of *Chasmosaurus* (up to 997 mm—CMN 8800), but exceeds that of *Vagaceratops* (up to 700 mm—CMN 41357). The infratemporal process projects anteriorly beyond the midpoint of the lateral temporal fenestra (Fig. 11), suggesting that the quadratojugal was excluded from the fenestra.

There are five variably preserved episquamosals on the left squamosal, and six on the right (Figs. 11A and 11B). Based on the extrapolated original length of the squamosals, and the spacing frequency of the preserved episquamosals, we estimate that each element probably had seven or possibly eight episquamosals. They are anteroposteriorly wide-based, low, and roughly equal in size (between 50 and 80 mm long) except for the anterior-most one, which is slightly longer (89 mm) with a more pronounced triangular shape. The squamosal-episquamosal sutures become increasingly more obvious posteriorly, as in other chasmosaurines (e.g., *Chasmosaurus*, Campbell et al., 2016: Fig. 9), but unlike centrosaurines, where episquamosals fuse in a posterior to anterior direction (Sampson, Ryan & Tanke, 1997).

**Parietal**: Most of the parietal is preserved (Figs. 10A and 10B), missing only the posterior bar and most of the lateral bars. The robust median bar (Figs. 10A and 10B) ranges in dorsoventral thickness from approximately 66 mm near the anterior end of the parietal fenestrae, to approximately 40 mm at its posterior-most preserved end. The posterior end of the bar is nearly square in cross-section and slightly laterally expanded, where it begins to diverge laterally into the posterior bar. The lateral margins of the median bar each have a wide, longitudinal groove that is mediolaterally shallow on the left side, but deep on the right side (approximately 15 mm at the deepest point). These grooves are not as pronounced as those of the *Chasmosaurus* specimen CMN 8803, in which the deep grooves create an I-beam cross-section for the median bar. Longrich (2010) considered an I-beam cross-section diagnostic of *Mojoceratops perifania* (CMN 8803), however, this character is absent in the holotype TMP 1983.025.0001 and referred specimen AMNH 5656, making the utility of this character questionable.

The parietal fenestrae are approximately 410 mm in anteroposterior length, as preserved, but most likely would have been longer, as the posterior bar is not preserved. The preserved length is within the range known for *Chasmosaurus* (354 mm on AMNH 5402 to 627 mm on NHMUK R4948), but greatly exceeds those reported for *Vagaceratops* (260 mm on TMP 1987.045.0001 to 265 mm on CMN 41357).

The parietal of CMN 34829 has a few notable asymmetries. One of these is a relatively large accessory fenestra situated on the right side of the median platform (Figs. 10A and 10B). This fenestra is nearly circular in shape with a smooth, non-swollen, rounded margin, measuring approximately 101 mm long and 82 mm wide, with vascular grooves continuing up to the edges. Accessory parietal fenestrae are exceedingly rare in chasmosaurine skulls, only known in *Anchiceratops ornatus* (UW 2419; Mallon et al., 2011) and *T. utahensis* (USNM 15583; Farke, 2010; see “Discussion”). The median platform also bears a large protuberance (Figs. 10A and 10B) that laterally projects almost halfway across the left dorsal temporal fenestra. This type of feature is unknown in *Chasmosaurus* or *Vagaceratops*; however, irregular bone growth of the skull roof is known in *Chasmosaurus* (e.g., the accessory bridge of bone over the frontoparietal fontanelle in CMN 8800 and YPM 2016).

### Braincase

The braincase is visible only in anterior and lateral views (Figs. 10 and 12), as the posteroventral region (e.g., occipital condyle, paroccipital processes) is inaccessibly embedded in matrix and plaster. It is almost completely preserved, but has undergone some fracturing and crushing, and the endocranium, nerve and arterial canals are infilled with matrix. As its morphology compares well with that of CMN 8802, only features that augment the description of the latter will be discussed below.

The distal end of each basipterygoid process is preserved, having a tapered but rounded tip and an expansive facet to receive the non-preserved pterygoid (Fig. 12). Unlike CMN 8802, the portion of the braincase anterior to the exit for the ophthalmic branch of the trigeminal nerve (cranial nerve V1) is preserved. Anteroventral to this nerve exit, the margins of the exit for the oculomotor nerve (cranial nerve III; MD approximately 12 mm) can be discerned, and anterior to that, the exit for the optic nerve (cranial nerve II; MD approximately 12 mm) (Figs. 12C–12F; Witmer & Ridgely, 2008). Ventral to the cranial nerve III exit lies the exit for the sphenoid artery canal (MD approximately 6 mm; Witmer & Ridgely, 2008).

The region ventral to the sphenoid artery canal where the exit for cranial nerve VI would be expected (Witmer & Ridgely, 2008) is obscured by matrix, as is the exit for the trochlear nerve canal (cranial nerve IV). The laterosphenoid buttress is better preserved than in CMN 8802, and includes the anterior knob, which appears to insert into the medial surface of the postorbital (Figs. 12C and 12D; Godfrey & Holmes, 1995).

The exit for the olfactory nerve (cranial nerve I) is laterally crushed, but visible in anterior view and situated on the midline of the anterior end of the braincase (Figs. 12C and 12D; Witmer & Ridgely, 2008). This exit is presumably surrounded by the paired laterosphenoids (Forster, 1996), but the sutures cannot be discerned and likely closed.

### Description of TMP 2011.053.0046

**Figure 13:**
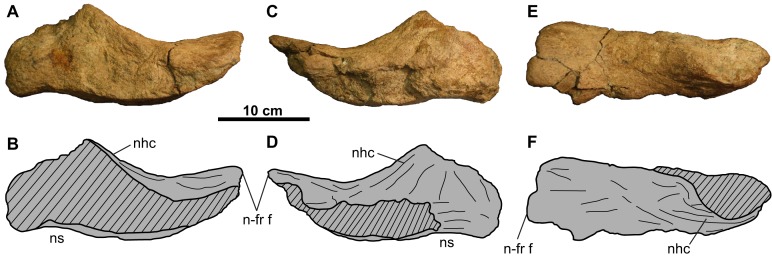
Nasal of TMP 2011.053.0046 (*Vagaceratops* sp.). Nasal in (A–B) left lateral, (C–D) right lateral, and (E–F) dorsal views. Hashed areas represent broken bone. For abbreviations, see Anatomical Abbreviations.

**Figure 14:**
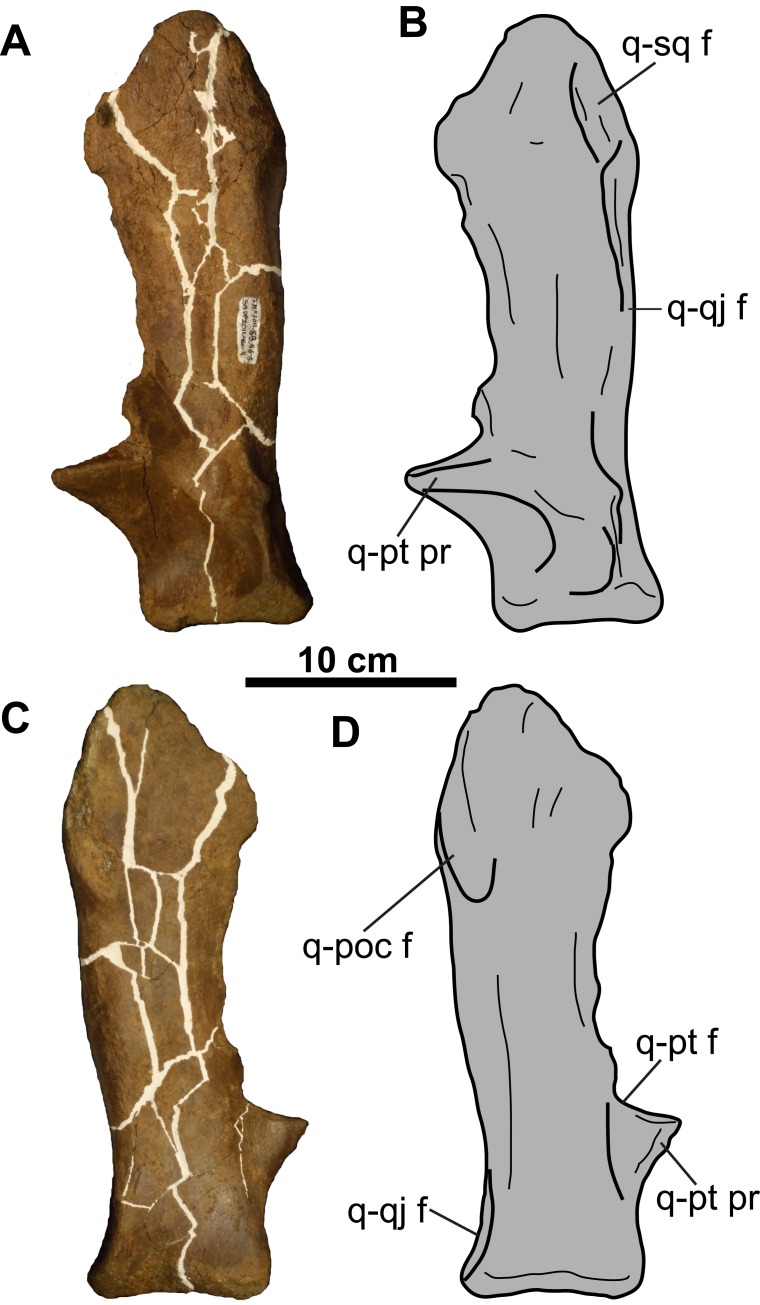
Left quadrate of TMP 2011.053.0046 (*Vagaceratops* sp.). Quadrate in (A–B) anterior and (C–D) posterior views. For abbreviations, see Anatomical Abbreviations.

**Figure 15:**
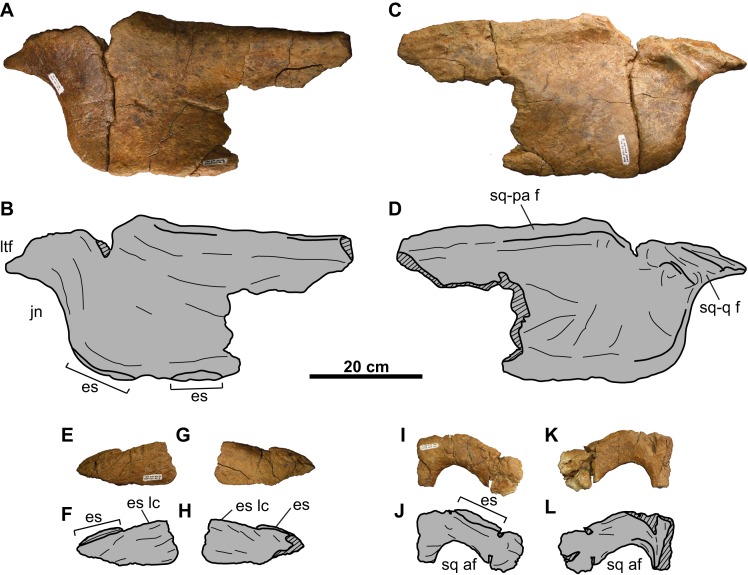
Left squamosal of TMP 2011.053.0046 (*Vagaceratops* sp.). Squamosal in (A–B) dorsal and (C–D) ventral views. Second squamosal fragment in (E–F) dorsal and (G–H) ventral views. Third squamosal fragment in (I–J) dorsal and (K–L) ventral views. Hashed areas represent broken bone. For abbreviations, see Anatomical Abbreviations.

**Figure 16:**
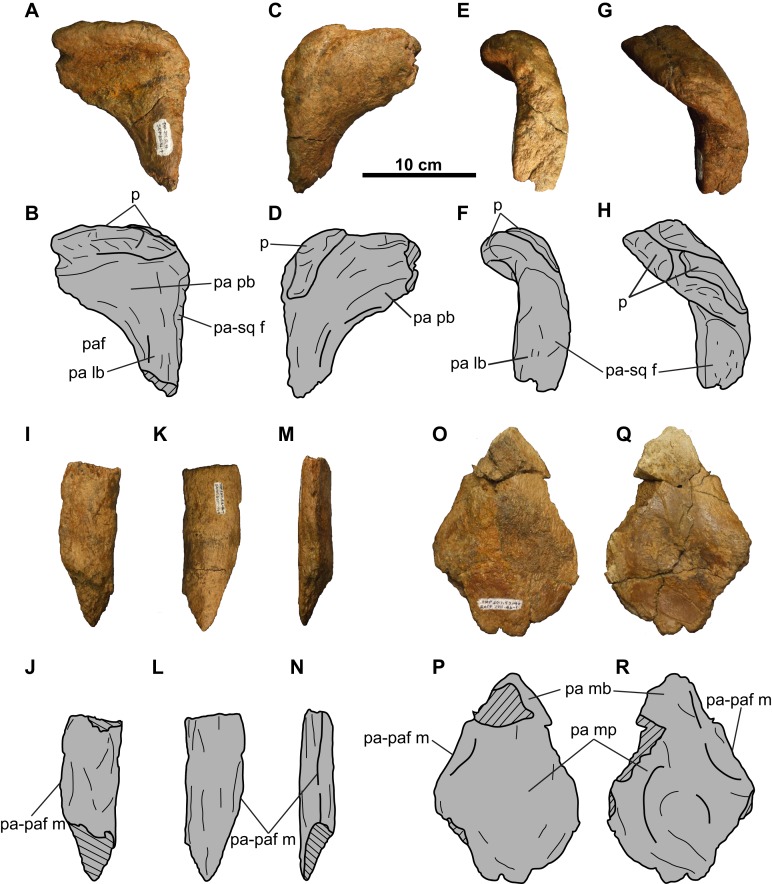
Parietal of TMP 2011.053.0046 (*Vagaceratops* sp.). Posterior bar fragment in (A–B) dorsal, (C–D) ventral, (E–F) lateral, and (G–H) posterolateral views. Lateral bar fragment in (I–J) dorsal, (K–L) ventral, and (M–N) medial views. Median platform fragment in (O–P) dorsal and (Q–R) ventral views. Hashed areas represent broken bone. For abbreviations, see Anatomical Abbreviations.

TMP 2011.053.0046 (Figs. 13–16) is a disarticulated, partial skull found and collected by DCE during the 2011 Southern Alberta Dinosaur Project field season, and prepared at the ROM. It was collected in the Milk River region, approximately 14.5 km southeast of Manyberries (Fig. 1), within 10 m of the base of the LCZ; precise locality information on file at the TMP. The specimen consists of most of the nasals, the left quadrate, most of the left squamosal, and fragments of the parietal.

### Snout region

**Nasal**: Parts of the coossified nasals are preserved, but composed mostly of the right element (Fig. 13). The anterior and descending lateral processes are not preserved. A mediolaterally elongate, convex facet for the frontal is preserved on the posterior-most end.

The dorsal rim of the external nares are preserved (Fig. 13). The base of a large horncore is centered posterior to the midpoint of the nares, as in both *Chasmosaurus* (e.g., CMN 2280, ROM 843) and *Vagaceratops* (CMN 41357, TMP 1987.045.0001). The anterior and dorsal half of the horncore is missing, so its original height and cross-sectional shape cannot be estimated. No epinasal can be discerned, but this may be due to incompleteness or advanced coossification.

### Cheek region

**Quadrate**: The preserved left quadrate (Fig. 14) is missing most of the pterygoid flange, but is otherwise complete. The remains of the pterygoid facet lie at the base of this flange, immediately dorsal to the pterygoid process. The lateral margin bears an elongate facet for the quadratojugal. Above this facet, on the anterior surface, is the facet for the squamosal (Figs. 14A and 14B). The paroccipital process is situated posterior to this latter facet (Figs. 14C and 14D). The ventral end is bilobate and approximately 82 mm wide.

### Parietosquamosal frill

**Squamosal**: The anterior half of the anteroposteriorly elongate, left squamosal is preserved (Figs. 15A–15D). The squamosal infratemporal process extends anteriorly and forms the ventral margin of the lateral temporal fenestra. An elongate facet to receive the quadrate is present on the medial surface, immediately above the jugal notch (Figs. 15C and 15D). Posteriorly, the ventromedial margin bears a facet for the lateral parietal bar. There are two equal-sized and low-profile episquamosals, whose sutures with the underlying lateral margin of the squamosal cannot be discerned (Figs. 15A–15D).

Two other squamosal fragments are preserved. The first (Figs. 15E–15H) represents part of the lateral margin with one articulated episquamosal and a neighboring locus for a second one, which was likely present in life but fell off post-mortem. The lateral surface of this fragment is rugose and vascularized (Figs. 15E and 15F), while the medial surface is smooth (Figs. 15G and 15H).

Similarly, the second fragment (Figs. 15I–15L) is thought to pertain to the lateral margin as noted by what appears to be an episquamosal on its lateral margin. The medial margin is concave in dorsal view, and represents what appears to be the lateral margin of an accessory fenestra. The fenestra margins do not appear to be irregular in growth, and vascular grooves continue up to the edge.

**Parietal**: The parietal (Fig. 16) is represented by three fragments: the left posterolateral corner; a short segment of the lateral bar; and the anterior end of the median bar and platform.

The posterolateral corner fragment (Figs. 16A–16H) marks the transition from the lateral bar to the posterior bar. The posterior bar portion is anteroposteriorly broad but dorsoventrally compressed, while the lateral bar portion is dorsoventrally thick but mediolaterally narrow. On the lateral margin of the lateral bar is the facet for the squamosal (Figs. 16E–16H), indicating that the squamosal extended almost as far posteriorly as the parietal. The posterior bar is relatively thin anteriorly, but thickens into an anterodorsally-recurving posterior margin. This recurved margin is situated immediately adjacent to the lateral margin of the parietal, similar to that seen in *Vagaceratops* (CMN 41357, TMP 1987.045.0001, and TMP 1998.102.0008; Campbell et al., 2016: Figs. 3P–3R), but unlike *Chasmosaurus* (e.g., AMNH 5656, CMN 2280, TMP 1983.025.0001).

The recurved posterior margin is adorned by what appear to be two well-articulated EPs, whose borders are difficult to delineate (Figs. 16A–16H). The suture for the medial-most EP is obliterated anteriorly, but can be faintly discerned on the posterior side (Figs. 16G and 16H). Lateral to this EP is a smooth, better defined, bulbous mass representing the basal remnants of a second EP. Based on its position and morphology, this second EP would most likely have been oriented posterolaterally.

The lateral bar segment (Figs. 16I–16N) is anteroposteriorly elongate. The dorsal sutural surface for the squamosal is rugose (Figs. 16I and 16J), while the ventral surface is smooth (Figs. 16K and 16L). The fragment tapers from a rounded lateral margin towards what probably represents the lateral margin of the parietal fenestra (Figs. 16M and 16N). Due to incomplete preservation, it cannot be determined whether the lateral bar excluded the squamosal from the lateral margin of the parietal fenestra.

The third fragment (Figs. 16O–16R) represents the anterior end of the median bar and part of the median platform. The dorsal surface is rugose and slightly convex (Figs. 16O and 16P), while the ventral side is smoother and gently concave (Figs. 16Q and 16R).

## Discussion

### CMN 8802

CMN 8802 is diagnosed as chasmosaurine based on its possession of a rostral with anteroposteriorly elongate ventral processes, no ventral expansion of the posteroventral margin of the premaxilla, long triangular squamosals with a lap joint for the parietal, and a transversely narrow and strap-like parietal median bar.

Among the chasmosaurine genera currently documented from the DPF (*Chasmosaurus*, *Vagaceratops*, *Mercuriceratops*, and possible remains of *Spiclypeus*) and age-equivalent sediments of the Oldman Formation in Alberta, CMN 8802 bears a closest resemblance to *Chasmosaurus*, as it is within the range of variation of this genus in all respects (Campbell et al., 2016). Although incomplete, the parietal fenestrae of CMN 8802 (Fig. 6) are quite elongate relative to those of *Vagaceratops* (CMN 41357 and TMP 1987.045.0001). CMN 8802 cannot be referred to *Mercuriceratops* (Ryan et al., 2014), as the latter has a hatchet-shaped squamosal morphology. CMN 8802 also lacks the relatively wide median parietal bar inferred for *Spiclypeus* (Mallon et al., 2016). The postorbital horncores of CMN 8802 are also unlike those of specimens previously referred to “*Mojoceratops*” (i.e., AMNH 5401, CMN 1254, CMN 34832, TMP 1979.011.0147, TMP 1981.019.0175, TMP 1983.025.001, and UALVP 40), which are significantly longer and have more pointed apices.

Campbell et al. (2016) diagnosed *Chasmosaurus* based on the following unique combination of characters: (1) Premaxillary flange along entire anterior margin of external naris; (2) postorbital horncores, when present, curve posteriorly along their length; (3) squamosal dorsal border laterally adjacent to dorsal temporal fenestra straight in profile, anteriorly at level with base of postorbital horncore, and sloping posteroventrally at a shallow angle before ascending farther posteriorly to form lateral border of parietal fenestra; (4) medial margin of squamosal, where it articulates with the lateral bar of the parietal, straight; (5) frill broadens posteriorly to form rectangular to triangular shield with maximum width more than twice the skull width at orbits; (6) parietal fenestrae large, occupying most of the parietal, and being rounded or anteroposteriorly longer than transversely wide; and (7) EPs straight and triangular in shape and oriented posteriorly or anterodorsally. None of these characters represent autapomorphies, as they are present in other chasmosaurines, but this combination appears to be unique to *Chasmosaurus*.

Characters 1, 2, and 7 cannot be confirmed in CMN 8802. The postorbital horncores of CMN 8802 may have undergone remodeling; if so, the original shape of the horncores (character 2) cannot be determined. Characters 3–6 are present in CMN 8802, although character 5 is reasonably inferred for this specimen. These four combined characters are absent in all chasmosaurines except for *Chasmosaurus*, *Pentaceratops*, and *Utahceratops*. However, CMN 8802 lacks the hyper-elongate epijugals that characterize *Pentaceratops* (Lehman, 1998). CMN 8802 also lacks the anteroposteriorly elongate middle episquamosals that characterize *Utahceratops* (Sampson et al., 2010). CMN 8802 is therefore tentatively referred to *Chasmosaurus*.

The original assignment of CMN 8802 as the paratype of *C. russelli* by Sternberg (1940) was based on his original diagnosis for this species: “skull large, relatively high and short in front of orbits; rostral straight inferiorly, not hooked downward at tip; nasal horncore massive; broad between orbits; no brow horncores; well-developed epijugal; parietals deeply indented posteriorly; squamosal border not strongly scalloped; epoccipitals small; mandible massive” (Sternberg, 1940: 478). The presence of an indented or embayed posterior parietal bar is the only one of the above features that has remained part of the diagnosis for *C. russelli* (Maidment & Barrett, 2011; Campbell et al., 2016), but the posterior bar is not preserved in CMN 8802 and therefore the original designation as the paratype of *C. russelli* by Sternberg (1940) is questionable.

Based on the above observations, CMN 8802 is tentatively referred to *Chasmosaurus* sp. If CMN 8802 is indeed referable to this genus, then this specimen represents one of the largest yet described. The occipital condyle measures 76 mm across, which is comparable to that of the largest described *Chasmosaurus* skull ROM 843 (79 mm; skull length approximately 200 cm). Also, the width of the squamosal (436 mm, including anterior-most episquamosal) is larger than that of ROM 843 (395 mm, including anterior-most episquamosal). CMN 8802 also represents the first occurrence of *Chasmosaurus* from the Oldman Formation (Campbell et al., 2016). However, the sediments from which CMN 8802 were collected are age-equivalent to those of the upper DPF, as exposed in DPP, and so CMN 8802 is within the stratigraphic range of other *Chasmosaurus* skulls collected in DPP (Fig. 1).

### CMN 34829

CMN 34829 is referred to Chasmosaurinae based on the following features: supracranial sinuses walled mostly by the postorbitals, and presence of elongate squamosals with a lap joint for the parietal. Characters 1, 2, and 7 for *Chasmosaurus* (Campbell et al., 2016) cannot be confirmed in CMN 34829. Like CMN 8802, the postorbital horncores of CMN 34829 may have also undergone remodeling. Characters 3–6 are present in CMN 34829, and this specimen can be distinguished from *Pentaceratops* and *Utahceratops* for the same reasons as CMN 8802 above. CMN 34829 is therefore tentatively referred to *Chasmosaurus* sp.

### TMP 2011.053.0046

TMP 2011.053.0046 is also assigned to Chasmosaurinae based on its inferred long triangular squamosals with a lap joint for the parietal, and an anteroposteriorly narrow and strap-like parietal posterior bar. The presence of an anterodorsally-recurved posterior parietal margin in TMP 2011.053.0046 is also present in mature specimens of *Chasmosaurus* and *Vagaceratops*. However, the anterodorsally-recurved ridge in TMP 2011.053.0046 is situated almost directly adjacent to the parietosquamosal contact, as in *Vagaceratops* (e.g., TMP 1998.102.0008; Campbell et al., 2016: Fig. 3P). Also, the two preserved EPs in TMP 2011.053.0046 are situated in similar positions as EPs 4 and 5 in *Vagaceratops*. This skull was also collected relatively high in section within the DPF, within 10 m of the base of the LCZ, placing it within the same stratigraphic interval as *Vagaceratops* (Campbell et al., 2016). For these reasons, TMP 2011.053.0046 is referred to *Vagaceratops* sp.

### Update on the distribution of accessory frill fenestrae in Chasmosaurinae

**Figure 17:**
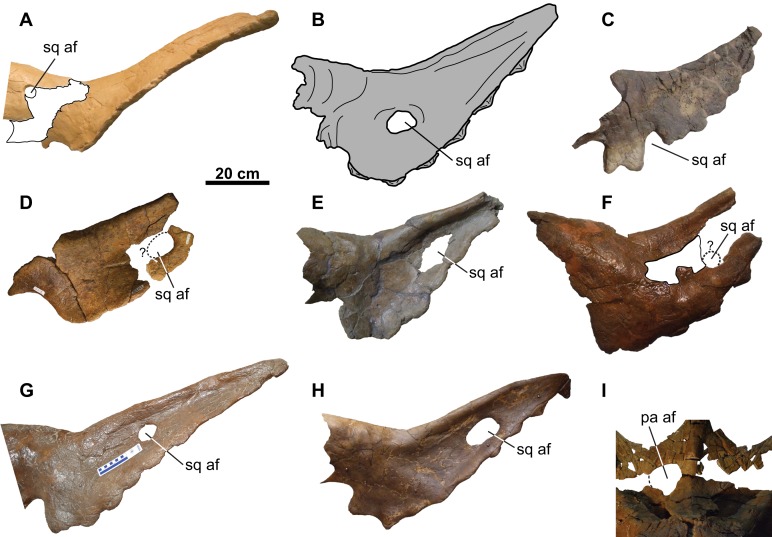
Distribution of accessory fenestrae in the parietosquamosal frill of chasmosaurines from the Dinosaur Park Formation and age-equivalent sediments of Alberta. (A) Lateral view of right squamosal of CMN 41357 (*Vagaceratops irvinensis* holotype, cast; image flipped). (B) Ventral view of left squamosal of NHMUK R4948 (traced from Fig. 8E of Maidment & Barrett, 2011; *Chasmosaurus belli*; image flipped). Dorsal view of left squamosal of (C) TMP 1987.045.0001 (*V. irvinensis*), (D) TMP 2011.053.0046 (*Vagaceratops* sp.), (E) TMP 1983.025.0001 (*Chasmosaurus russelli*), (F) CMN 8802 (*Chasmosaurus* sp.), (G) ROM 843 (*C. belli*; photo credit: Jordan Mallon), and (H) ROM 839 (*Chasmosaurus* sp.). (I) Dorsal view of anterior portion of parietal of CMN 34829 (*Chasmosaurus* sp.). Dotted lines in (D), (F), and (I) represent inferred margins of accessory fenestrae. For abbreviations, see Anatomical Abbreviations.

Accessory squamosal fenestrae such as those in CMN 8802 and TMP 2011.053.0046 are not uncommon amongst other chasmosaurine skulls from the Belly River Group of Alberta (i.e., *Chasmosaurus* skulls NHMUK R4948, ROM 839, ROM 843, and TMP 1983.025.0001; *Vagaceratops* skulls CMN 41357 and TMP 1987.045.0001; Fig. 17). Interestingly, in all these specimens except CMN 41357 (Fig. 17A), the fenestrae occur on the left squamosal. The position of the fenestra in TMP 2011.053.0046 is uncertain, as the fragment preserving the fenestra margin may or may not belong to the left side of the skull (Fig. 17D). Tanke & Farke (2007) were the first to recognize that squamosal fenestrae in chasmosaurines occur more frequently on the left side of the frill, noting their occurrence in *Chasmosaurus*, as well as *Arrhinoceratops brachyops* (ROM 796), and *Pentaceratops sternbergii* (PMU R200). Accessory fenestrae also occur on the left squamosal of *Utahceratops gettyi* (UMNH VP 16784; Sampson et al., 2010) and *S. shipporum* (CMN 57081; Mallon et al., 2016). Fenestrae are known to occur less commonly on the right squamosal in chasmosaurines, (*Vagaceratops* [CMN 41357] and *Triceratops* cf. *T. prorsus* [RSM P1163.4]; Tanke, Farke & Gilbert, 2009), and are often associated with a left squamosal fenestra as well (USNM 2412, *Nedoceratops hatcheri*; MPM VP8149, *T. latus*; EM P16.1, *Torosaurus* cf. *T. latus*; (Marshall & Barreto, 2001; Tanke & Farke, 2007)). The predominance of fenestrae on the left (*n* = 14) versus the right (*n* = 5) side is based on a limited sample size, but this could be tested further with the discovery of more specimens.

Tanke & Farke (2007) also observed that, although the position of fenestrae within the squamosal is variable (Fig. 17), they do not occur directly adjacent to the lateral or medial margins. However, Tanke, Farke & Gilbert (2009) later reported on a skull (RSM P1163.4, *Triceratops* cf. *T. prorsus*) with a very large accessory fenestra incising the medial portion of the right squamosal and lateral parietal region. Mallon et al. (2016: Fig. 8D) reported on a mediolaterally-arranged series of three fenestrae on the left squamosal of CMN 57081 (*S. shipporum*), with the lateral-most one situated between the second and third anterior-most episquamosals. Another notable case is the left squamosal of TMP 1987.045.0001 (*Vagaceratops*), which has a distinctive notch also situated between the second and third anterior-most episquamosals, and penetrating deep into the body of the squamosal (Fig. 17C). Holmes et al. (2001) noted that this notch was unique among ceratopsids, but they did not offer an explanation to account for its presence. The location of this notch compares well with the condition in CMN 57081. Perhaps this notch began as one or more squamosal fenestrae, as in CMN 57081, but later expanded and resorbed the lateral squamosal margin and possibly an episquamosal as well.

Tanke & Farke (2007) proposed that most squamosal fenestrae in chasmosaurines appear to develop via non-pathological bone resorption, due to the apparent lack of any signs of trauma or disease in the specimens they examined. They did, however, note that the margins of the fenestra on the left squamosal of USNM 2412 (*N. hatcheri*) are swollen, irregular in shape, and have an irregular vascularized surface texture, which they interpreted as being due to a traumatic injury. Such an interpretation was also made by Marshall & Barreto (2001) for a similar fenestra on the left squamosal of MPM VP8149 (*T. latus*). Tanke, Farke & Gilbert (2009) suggested that the large accessory parietosquamosal fenestra on the right side of the skull of RSM P1163.4 (*Triceratops* cf. *T. prorsus*) may have began as a smaller perforation incurred by a conspecific horncore thrust, and later expanded outwards; however, they also left open the possibility that this fenestra may have had a non-pathological origin. Mallon et al. (2016) also suggested that the fenestrae in the left squamosal of CMN 57081 (*S. shipporum*) are pathological in origin, based on their association with what appear to be abscess cavities and drainage tracts. Mallon et al. (2016) postulated that these fenestrae were created by a horncore thrust from a conspecific and later became infected, resulting in chronic osteomyelitis. None of the squamosal fenestrae in the skulls of *Chasmosaurus* and *Vagaceratops* (Fig. 17) have such irregular bone growth, and are tentatively considered herein to be non-pathological in origin (sensu Tanke & Farke, 2007).

Accessory parietal fenestrae are quite rare in ceratopsids, and have been previously reported only in the chasmosaurines *A. ornatus* (UW 2419; Mallon et al., 2011: Fig. 9A) and *T. utahensis* (USNM 15583; Farke, 2010: Fig. 6). UW 2419 has two small accessory fenestrae (measuring 80 mm long and 51 mm wide, and 43 mm long and 27 mm wide, respectively) on the anterior end of the left side of the parietal, (Tanke & Farke, 2007; Mallon et al., 2011), similar in position but smaller than that of CMN 34829 (Figs. 10A and 10B). USNM 15583 has a small accessory foramen along the medial margin of the anterior end of the left dorsotemporal fenestra (Farke, 2010). It cannot be determined whether this foramen perforated the floor of the parietal (forming a fenestra), or if it was floored by bone, as this area is obscured with plaster.

Most of the accessory fenestra in RSM P1163.4 (*Triceratops* cf. *T. prorsus*; Tanke, Farke & Gilbert, 2009) is situated within the body of the squamosal, where it is thought to have originated, and only later expanded into the parietal. *N. hatcheri* (USNM 2412) has a narrow, irregular-shaped fenestra (133 mm long by 50 mm wide) on the right side of the parietal, where the normal parietal fenestra would be expected (Farke, 2011); the left side of the parietal is insufficiently preserved to tell whether this side also had a fenestra. The fenestra in *N. hatcheri* is considered by Lull (1933) and Longrich & Field (2012) to be pathological in origin (i.e., an accessory fenestra), based on its irregular shape, while Farke (2011) and Scannella & Horner (2011) believe it represents a small, normal parietal fenestra, as the margins are not swollen (Farke, 2011; Scannella & Horner, 2011). Farke (2011) considers *N. hatcheri* as representing a valid taxon, based on a suite of characters that are unique to USNM 2412. Scannella & Horner (2011) postulate that the parietal of USNM 2412 may represent a transitional, ontogenetic stage between the unfenestrated and fenestrated parietals of *Triceratops* and *Torosaurus*, respectively, representing a prolonged ontogenetic sequence in *Triceratops*. Longrich & Field (2012) did not find support for the synonymy of *Triceratops* and *Torosaurus*, but instead refer USNM 2412 to *T. horridus*.

The bone surrounding the accessory fenestra in the median parietal platform of CMN 34829 is smooth with no evidence of swelling, and is tentatively considered herein to be non-pathological in origin (sensu Tanke & Farke, 2007). This part of the parietal is relatively thin, like the center of the squamosal, where an accessory fenestra is likely the easiest to develop.

## Conclusion

This study reports upon two chasmosaurine skulls from the Campanian DPF (CMN 34829 and TMP 2011.053.0046) and one from the Oldman (CMN 8802) Formation of Alberta, Canada. CMN 8802 and CMN 34829 are both tentatively referred to *Chasmosaurus* sp. If the taxonomic identification for CMN 8802 is correct, this effectively expands the genus *Chasmosaurus*, previously known only from the DPF, into the uppermost sediments of the Oldman Formation, which, in southernmost Alberta, are age-equivalent to the uppermost DPF as exposed in DPP. TMP 2011.053.0046 is tentatively referred to *Vagaceratops* sp.

Each of these three skulls has an accessory fenestra in either the squamosal (CMN 8802 and TMP 2011.053.0046) or parietal (CMN 34829). Squamosal fenestrae are fairly common in other skulls of *Chasmosaurus* and *Vagaceratops*, but the presence of an accessory parietal fenestra in a ceratopsid skull is exceedingly rare. The accessory fenestrae in these three skulls are tentatively considered to be non-pathological in origin.

## Anatomical Abbreviations

I: olfactory nerve canal
II: optic nerve canal
III: oculomotor nerve canal
V_1_: trigeminal nerve canal, ophthalmic branch
V_2&3_: trigeminal nerve canal, maxillary and mandibular branches
VI: abducens nerve canal
VII: facial nerve canal
VIII: vestibulocochlear nerve canal
IX: glossopharyngeal nerve canal
X-XI: shared canal for vagus (cranial nerve X) and accessory (cranial nerve XI) nerves
X-XII: shared canal for vagus (cranial nerve X) and accessory (cranial nerve XI) nerves, and anterior-most canals of hypoglossal nerve (cranial nerve XII)
XII: hypoglossal nerve canal
a: angular
add: adductor fossa
ar: articular
bo: basioccipital
bpt pr: basipterygoid process
bsph: basisphenoid
bsph-pt f: pterygoid facet on basisphenoid
bt: basal tuber
car: cerebral carotid artery canal
cpr: coronoid process
crp: crista prootica
crt: crista tuberalis
d: dentary
d-d f: dentary facet on dentary
d-pd f: predentary facet on dentary
dtf o: opening of the dorsal temporal fenestra
eo: exoccipital
epij: epijugal
es: episquamosal
es lc: episquamosal locus
exo-so f: supraoccipital facet on exoccipital
f: foramen
fm: foramen magnum
fo: fenestra ovalis
fpf: frontoparietal fontanelle
fr: frontal
fr-n f: nasal facet on frontal
fr-prf f: prefrontal facet on frontal
gle: glenoid fossa
ica: internal carotid artery
j: jugal
j-m f: maxilla facet on jugal
jn: jugal notch
lsph: laterosphenoid
lsphb: laterosphenoid buttress
ltf: lateral temporal fenestra
n-fr f: frontal facet on nasal
nhc: nasal horncore
ns: nares
oc: occipital condyle
op: opisthotic
orb: orbit
p: epiparietal
pa: parietal
pa af: accessory fenestra in parietal
paf: parietal fenestra
pa lb: lateral bar of parietal
pa mb: median bar of parietal
pa mp: median platform of parietal
pa mp prt: protuberance on median platform of parietal
pa-paf m: margin of parietal fenestra on parietal
pa pb: posterior bar of parietal
par: prearticular
pa-sq f: squamosal facet on parietal
pm: premaxilla
pm-m f: maxilla facet on premaxilla
pm-n f: nasal facet on premaxilla
pm s: premaxillary septum
po: postorbital
poc: paroccipital process
poc-q f: quadrate facet on paroccipital process
pohc: postorbital horncore
po-j f: jugal facet on postorbital
po-pa f: parietal facet on postorbital
po-ppb f: palpebral facet on postorbital
po-sq f: squamosal facet on postorbital
p-pa f: parietal facet on epiparietal
ppb: palpebral
prt: prootic
q: quadrate
qj: quadratojugal
q-poc f: paroccipital process facet on quadrate
q-pt f: pterygoid facet on quadrate
q-pt pr: pterygoid process of quadrate
q-qj f: quadratojugal facet on quadrate
q-sq f: squamosal facet on quadrate
r: rostral
r vpr: ventral process of rostral
sa: surangular
scs: supracranial sinus
scs m: margin of supracranial sinus
spha: sphenoid artery canal
spl: splenial
sq: squamosal
sq af: accessory fenestra in squamosal
sq-es f: squamosal facet on episquamosal
sq-pa f: parietal facet on squamosal
sq-poc f: paroccipital facet on squamosal
sq-po f: postorbital facet on squamosal
sq-q f: quadrate facet on squamosal;
vls: ventral longitudinal sinus.

## Institutional Abbreviations

AMNH: American Museum of Natural History, New York, NY, USA
CMN: Canadian Museum of Nature, Ottawa, ON, Canada
EM: Eastend Museum, Eastend, SK, Canada
MPM: Milwaukee Public Museum, Milwaukee, WI, USA
NHMUK: Natural History Museum of the United Kingdom, London, UK
PMU: Paleontological Institute at Uppsala University, Uppsala, Sweden
ROM: Royal Ontario Museum, Toronto, ON, Canada
RSM: Royal Saskatchewan Museum, Regina, SK, Canada
TMP: Royal Tyrrell Museum of Palaeontology, Drumheller, AB, Canada
UALVP: University of Alberta, Laboratory for Vertebrate Palaeontology, Edmonton, AB, Canada
UCMP: University of California Museum of Paleontology, Berkeley, CA, USA
UMNH VP: Utah Museum of Natural History Vertebrate Paleontology Collections, Salt Lake City, UT, USA
USNM: National Museum of Natural History, Washington, D.C., USA
UW: University of Wyoming, Laramie, WY, USA
YPM: Yale Peabody Museum, New Haven, CT, USA.

## Additional Information and Declarations

### Field Study Permissions

The following information was supplied relating to field study approvals (i.e., approving body and any reference numbers): TMP 2011.053.0046 was collected under a Palaeontological Research Permit to DCE in 2011, provided by Alberta Culture and Community Spirit Heritage Division and the Royal Tyrrell Museum of Palaeontology.

### Data Availability

The following information was supplied regarding data availability: The specimens CMN 8802 and CMN 34829 are both stored at the Canadian Museum of Nature, and the specimen TMP 2011.053.0046 is stored at the Royal Tyrrell Museum of Palaeontology.
